# Have I Seen This Place Before? A Fast and Robust Loop Detection and Correction Method for 3D Lidar SLAM

**DOI:** 10.3390/s19010023

**Published:** 2018-12-21

**Authors:** Michiel Vlaminck, Hiep Luong, Wilfried Philips

**Affiliations:** Image Processing and Interpretation (IPI), imec research group at Ghent University, Department of Telecommunications and Information Processing (TELIN), Ghent University, Sint-Pietersnieuwstraat 41, 9000 Gent, Belgium; hiep.luong@ugent.be (H.L.); wilfried.philips@ugent.be (W.P.)

**Keywords:** loop detection, lidar, point clouds

## Abstract

In this paper, we present a complete loop detection and correction system developed for data originating from lidar scanners. Regarding detection, we propose a combination of a global point cloud matcher with a novel registration algorithm to determine loop candidates in a highly effective way. The registration method can deal with point clouds that are largely deviating in orientation while improving the efficiency over existing techniques. In addition, we accelerated the computation of the global point cloud matcher by a factor of 2–4, exploiting the GPU to its maximum. Experiments demonstrated that our combined approach more reliably detects loops in lidar data compared to other point cloud matchers as it leads to better precision–recall trade-offs: for nearly 100% recall, we gain up to 7% in precision. Finally, we present a novel loop correction algorithm that leads to an improvement by a factor of 2 on the average and median pose error, while at the same time only requires a handful of seconds to complete.

## 1. Introduction

Mapping an environment using mobile mapping robots is a topic that has been studied for almost two decades. Still, it can be considered as a highly active research area as the ultimate goal of lifelong mapping is far from reached. In the ideal case, lifelong mapping comprises a solution in which the map of the *world* is continuously updated at a pace equalling the one at which the world is changing itself. During the past years many techniques have been introduced that can perform incremental mapping using both regular cameras and depth sensing technologies based on either structured light, ToF, or pulsed lidar. As novel and more accurate sensors with increasing resolutions are continuing to come to the market, the performance of these mapping solutions is increasing. However, even though these sensing technologies are producing more accurate depth measurements, they are still far from perfect; as a result, the proposed solutions suffer—and will continue to suffer—from the drift problem. These drift errors could be corrected by incorporating sensing information taken from places that have been visited before. This requires both algorithms that can recognize revisited areas as well as algorithms that can close the loop and propagate errors back in the pose graph. Unfortunately, existing solutions for loop detection and closure for 3D data are computationally demanding. In this work, we focus on speeding up both loop detection and loop correction in scanning lidar data. We propose a technique that is able to automatically detect and correct loops in 3D lidar data in a highly efficient way, thereby exploiting the power of modern GPUs.

## 2. Related Work

Techniques to conduct loop detection in 3D data can roughly be categorized into three main classes: local keypoint detection and matching in combination with a bag-of-words (BoW) approach, global descriptor matching, and a remainder category based on geometric primitives or whole objects. The first class is generally detecting salient keypoints in a point cloud, computing signatures for these keypoint positions, building a BoW, and finally matching them in different scans [1]. Many keypoint detectors have been proposed in the literature, such as intrinsic shape signatures (ISSs) [2], Harris 3D [3], Sift 3D [4], NARF [5], as well as many descriptors such as spin images [6] and SHOT [7]. However, despite this abundance of choice, the detection of distinctive keypoints with high repeatability remains a challenge in 3D point cloud analysis.

One way of dealing with this lack of high repeatability is by using *global* descriptors, which usually come in the form of histograms: the *fast point feature histogram* (FPFH) [8] and the *viewpoint feature histogram* (VFH) [9] are a couple of examples. Recently, He et al. [10] presented a novel global 3D descriptor for loop detection, named *multiview 2D projection* (M2DP), that is very promising regarding both accuracy and efficiency. However, this global descriptor matching along with its local counterpart continues to suffer from respectively the lack of descriptive power or the struggle with invariance. As a result, many loops are not detected or too many false positives are present, which on its turn imposes a restriction on fast and reliable loop closure. More recently, researchers tend toward the application of convolutional neural networks (CNNs) to learn both the feature descriptors as well as the metric for matching them in a unified way [11,12,13,14]. A severe limitation of these methods on the other hand is that they need a tremendous amount of training data. Moreover, they do not generalize well when trained and applied on data with varying topographies or acquired under different conditions. A model that was trained on data originating from traffic environments might perform poorly on indoor scenes and vice versa.

Finally, a third group of methods focus on place recognition based on either complete objects or planes. In [15], Moral et al. presented a place recognition algorithm based on plane-based maps. Unfortunately, their approach is only suitable for indoor man-made environments and is not scalable to outdoor scenes. Dube et al. [16], on the other hand, proposed a method based on segments for which they compute several descriptors that are integrated in a learning framework. Before feeding the segment descriptors to the recognition model, they first subject them to a geometric verification test. As their method is relying on all kinds of segments, it is more scalable than the work of Moral et al. However, when used in man-made environments such as buildings, some scans (e.g., parts of corridors) might lack geometrical features and may jeopardize the loop detection. Besides the three aforementioned categories, there are also 3D lidar SLAM systems that only use visual information to detect and close the loop [17].

Regarding the actual loop correction, many solutions have been presented [18,19,20]. Unfortunately, a severe drawback of these methods is their high complexity and computational burden, especially when the optimal solution is sought for. Some algorithms therefore opt to conduct the loop closure in 2D, such as the work of Hess et al. [21] in which they propose a real-time loop closure algorithm for 2D lidar SLAM and which is part of Google’s cartographer. Another approach was taken in [22], where the authors propose a heuristic suitable for large-scale 6D SLAM. Their idea is to conduct an optimization without any iteration between the SLAM front- and back-end, yielding a highly efficient loop closing method. In this work, we decided to use the same strategy as we want to end up with an online—hence very fast—solution. In [23], the authors propose the adoption of a hierarchical approach instead by subdividing the 3D map into local sub-maps. In order to incorporate corrections, the individual 3D scans in the local map are modeled as a sub-graph and graph optimization is performed to account for drift and misalignments only at the level of the local maps.

## 3. Contributions

The loop closure method developed in this work is part of an entire 3D mapping system that was presented in [24] and [25]. As in that work, we use lidar data originating from Velodyne scanners, including the VLP-16, HDL-32E, and HDL-64E. One of the main contributions of [24] was a scan matching framework that aligns newly acquired point clouds with an online built 3D map. As stated in the introduction, this procedure is prone to error accumulation, and for that reason we improve it by actively detecting loops, i.e., locations that the robot or mobile platform is visiting more than once, in order to propagate the error back in the SLAM pose graph. The main contributions of this work can be summarized as follows:

1. We accelerated the computation of a global 3D descriptor to detect strong loop candidates in lidar data. Compared to many other 3D descriptors, ours does not depend on the estimation of surface normals in the point cloud. The main motivation for this is the high difficulty of estimating these normals accurately given the sparse and inhomogeneous point density of lidar point clouds. Our 3D descriptor thus leads to more robust loop detections. A compiled version of our algorithm has been made available to the community through a GitHub repository, allowing future use (https://github.com/Shaws/m2dp-gpu).
2. We propose a global registration technique based on four-point congruent sets, inspired by the work of Mellado et al. [26]. We improved the efficiency significantly by omitting its randomized component, which leads to faster execution times. Moreover, our improvements make the registration technique more robust for sparse and inhomogeneous 3D data.
3. We propose and evaluate a loop correction algorithm that omits the iteration between the SLAM front- and back-end, leading to very fast computations of the solution.

## 4. Approach

As mentioned in the previous section, our loop closure pipeline consists of two main steps. Prior to these two steps, we perform a quick selection of loop candidates by testing for each newly computed position as to whether or not it is *close* to a place we already visited. By assuming that the local registration is quite accurate, we set a threshold on the distance between two poses, equalling 10% of the total trajectory since the last loop closure. For instance, when we travelled for 100 m since the last loop closure, we consider a location to be matchable if its computed pose is within 10 m. Once such a potential loop is detected, the next step deals with the matching of the “start” and “end” point cloud. As briefly mentioned in the previous section, this matching is done using a *global* signature that we compute for each of the two point clouds. The signature is based on the projection of the point cloud on several 2D planes, similar to the method described in [10]. If the matching residual of this method is sufficiently low, we consider it a strong loop candidate.

**Figure 1:**
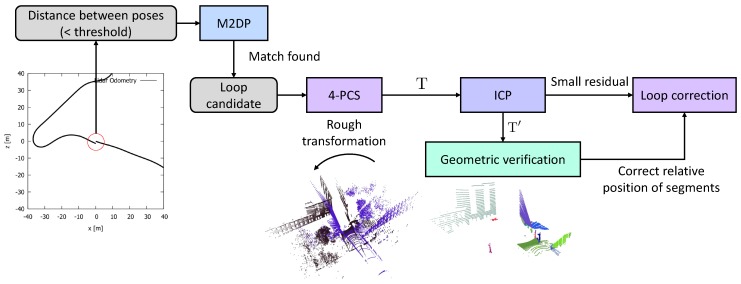
Our loop detection and correction pipeline, consisting of five main algorithms. The global descriptor MD2P detects loop candidates, after which the 4-PCS and ICP algorithm try to align both ends of the loop. Finally, the geometric verification step checks if the different segments in the scene are relatively still at the same position.

To guarantee that we are not dealing with a false positive, the next step seeks for the transformation that aligns the two point clouds. In case the loop candidate is a true positive, we expect the overlap of the two point clouds after registration to be very high. To this end, we adopt a *global* alignment technique, based on four-point congruent sets, to obtain a rough estimate of the transformation between the two ends of the loop. The alignment of the two ends is essential to eventually close the loop, as it can happen that the same position is revisited from an entirely different direction. Thus, the lidar scans can be acquired from different viewpoints. The use of an ICP-based method is in this case not appropriate as it will converge to a wrong local minimum. After the rough alignment, we still refine the transformation estimate using a variant of the ICP algorithm. After this step, we eventually do a quick geometric verification check to see if the objects in the two (transformed) point clouds are relatively still located at the same position. Figure 1 depicts a schematic overview to summarize our approach.

### 4.1. Multiview 2D-Projection

**Figure 2:**
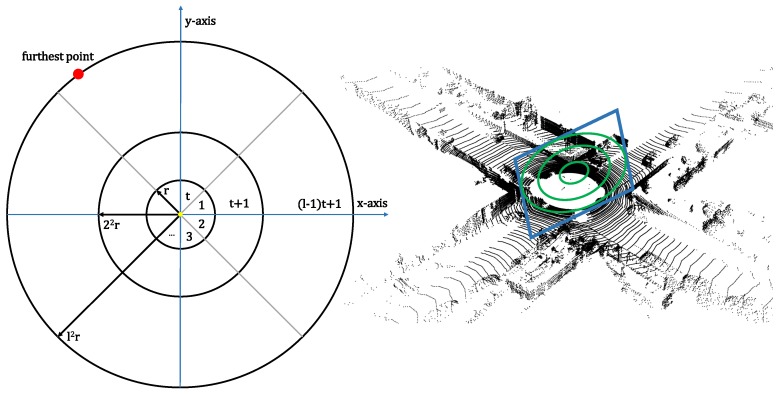
The generation of a 2D signature by projecting the 3D data onto a plane. The plane is divided into bins as follows: starting from the projected centroid, *l* concentric circles are generated and the maximum radius is set to the distance between the centroid and the furthest point. Each ring is subsequently subdivided into *t* bins, generating a lt×1 signature vector. Finally, the number of points lying in each bin are counted.

Our global point cloud descriptor is inspired by the method of multiview 2D-projection (M2DP), first presented by He et al. in [10]. The design of the descriptor is left unchanged, but we developed a novel implementation that exploits the GPU to its maximum. The algorithm is summarized as follows. In order to achieve rotation invariance, we first compute the centroid of the point cloud—denoted by P—and shift it toward this centroid to achieve zero mean for the points. Second, we define a reference frame by estimating two dominant directions of the point cloud using PCA. The two principal components are then set as the x- and y-axes of the descriptor reference frame. The third step consists of generating several 2D signatures by defining different planes on which we project the 3D data. Each plane can be represented using the *azimuth* angle θ and *elevation* angle ϕ. Thus, each pair of parameters [θ,ϕ] leads to a unique plane $X_{j}$ with normal vector $n_{j}={\left[\cos\theta \cos\phi ,\cos\theta \sin\phi ,\sin\theta \right]}^{⊤}$. The projection of a point $p_{i}\in P$ on $X_{j}$ is then given by $p_{i}^{j}=p_{i}-\frac{p_{i}^{⊤}n_{j}}{\left|\right|n_{j}{\left|\right|}_{2}^{2}}n_{j}$. To describe the structure of the points on $X_{j}$, the 2D plane is further divided into bins as follows (cfr. Figure 2). Starting from the projected centroid on $X_{j}$, *l* concentric circles are generated, with radii $\left[r,2^{2}r,l^{2}r\right]$ and the maximum radius is set to the distance of the centroid and the furthest point. Each ring is divided in *t* bins, hence defining l×t different bins. For every bin, the number of projected points lying in it are counted, generating a lt×1 signature vector $v_{j}$ describing the points projected on plane $X_{j}$.

The main benefit of this projection is that it is not relying on the estimation of surface normals for the points. This latter procedure is usually time-consuming and often times inaccurate for lidar data given their sparse and inhomogeneous point density. The signature is computed for *p* different azimuth angles and *q* different elevation angles where the stride on azimuth is $\frac{\pi }{p}$ and the one on elevation $\frac{\pi }{2q}$. Hence, there are pq different planes, leading to a signature matrix *A* of size pq×lt, for which each row corresponds with a single signature vector $v_{j}$. Finally, an SVD decomposition is run on the matrix *A* and the first left and right singular vectors are concatenated to form the final descriptor.

Many parts of this algorithm can be computed in parallel, allowing us to exploit the multi-core nature of modern GPUs. To ease the implementation, we made use of Quasar, a language and computing platform facilitating GPU programming that was presented in [27]. Specifically, three major parts were accelerated, the first one being the determination of the two dominant directions of the point cloud computed using PCA. Our PCA implementation uses a parallelized version of the SVD algorithm. The second part deals with the projection of the points on the different planes $X_{j},j=1,\ldots ,pq$, all of which can be computed in parallel. This leads to a speed up for this part of pq×N, *N* being the number of points in P, compared to a serial version of the algorithm. Finally, once the projections are computed for all planes and all points, the numbers of points belonging to each bin can also be determined in parallel.

### 4.2. Four-Point Congruent Sets

**Figure 3:**
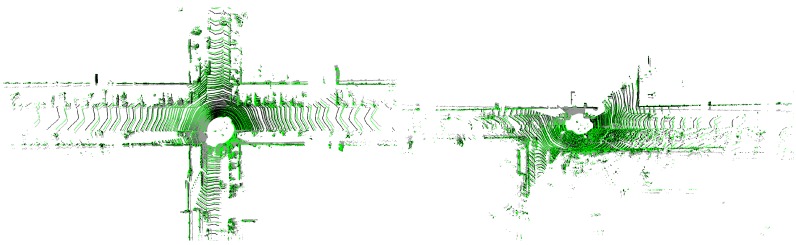
Two different examples of registered point clouds seen from a bird’s-eye view, originating from the KITTI ‘00’ sequence. The black and gray scans have been selected by the M2DP algorithm as the start and the end point of a potential loop. The two point clouds are, however, slightly rotated. Our registration technique aligns both (the green point cloud is the transformed version of the gray one) and determines their overlap. When the overlap is sufficiently high, the loop candidate is eventually selected as a true loop. Here, one can see that the green and black point cloud are sufficiently overlapping.

The algorithm described in the previous section provides loop candidates. However, its outcome is insufficient for the loop to close. As the descriptor is rotation-invariant, we need to find out the transformation between the two point clouds in order to *discard* it from the error back propagation. To this end, we need a registration algorithm that can deal with large variations in orientation. Figure 3 depicts a bird’s-eye view of the point clouds at both ends of the loop and demonstrates why the registration is necessary. The black and gray point clouds are respectively the start and end point of a loop. The green point cloud is the transformed version of the gray one. If after registration the overlap between the two point clouds (green and black) is sufficiently high, we accept it is a true loop.

Another key thing to keep in mind is that it can happen that a part of the scene has been changed in the meantime. Think about parked cars along the road that disappear or other ones that have taken their place. The use of local feature descriptors in a BoW approach would most likely fail in these cases. For that reason, our global registration technique takes into account the main geometry of the scene instead of relying on keypoint positions. The core of our registration technique is based on *four-point congruent sets* (4-PCS), an idea initially proposed by Aiger et al. [28] and later improved by Mellado et al. [26]. Our 4-PCS method is thus a global point cloud registration technique that does not rely on the extraction of features. Instead it is matching congruent sets in both point clouds, thereby adopting a *generate-and-test* paradigm, known from *random sample consensus* (RANSAC) solutions. In its most simple form, RANSAC randomly selects three points from both the source point cloud P and the target point cloud Q and subsequently computes the rigid transformation using these points. Next, it tries to *verify* this transformation by determining how many points from P are within a δ-distance from points in Q after applying the transformation. If this count—usually referred to as the size of the *consensus set*—is sufficiently high, the transformation is accepted as a good solution. Otherwise, the process is repeated by randomly selecting another triplet of points. The transformation with the largest consensus set is finally accepted as the *best fit*. The 4-PCS method builds on this randomized alignment approach, but instead of exhaustively testing all the triplets from Q, it introduces the concept of *planar congruent sets* to select only a small subset of bases from Q that can potentially match a given base from P. The first step in the 4-PCS method thus consists of selecting a four-point coplanar base *B* from the source point cloud P. Next, from the target point cloud Q, all four-point sets $\{U_{1},\ldots ,U_{N}\}=U$ that are approximately congruent to *B* are determined. Third, for all sets $U_{i}$, the rigid transformation $T_{i}$ that aligns *B* and $U_{i}$ is computed and verified according to the *largest common point set* (LCP) criterion. This latter criterion denotes the set of points $S_{i}\in P$ that are within δ-distance from a point in Q after applying the transformation. Finally, the best transformation $T_{opt}$, i.e., the one leading to the set $S_{k}$ with the highest cardinality, is kept. In summary, the aforementioned algorithm consists of four major steps: (1) selecting a coplanar base in one point cloud, (2) finding the (approximate) congruent sets in the second point cloud, (3) computing the rigid transformations, and (4) testing the rigid transformations and selecting the best one.

#### 4.2.1. Selecting a Coplanar Base

**Figure 4:**
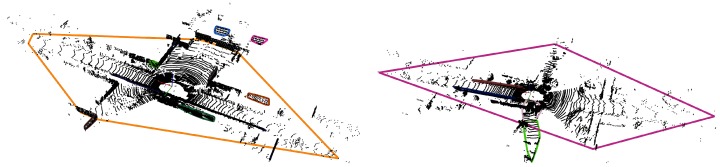
Two different examples showing how to select a coplanar base. First, several objects in the scene are clustered, after which they are subjected to a plane-fitting algorithm. In case a few planes have been detected, their convex hulls are computed. A coplanar base is then selected by picking four points close to the convex hull.

One of the main limitations of the original method by Aiger et al. [28] is its random search for coplanar points, which is leading to substantial unnecessary computational burden. Instead, we propose clustering the 3D points and subjecting them to a plane fitting process. As the point clouds are generated by a scanning lidar device with 16–64 colinear lasers, we can project the 3D laser points onto a regular 2D grid, as described in [24]. Doing so, we can exploit the known adjacency of the points to quickly perform clustering. More specifically, we adopt a region growing algorithm using two comparator functions to determine whether or not two neighboring points belong to the same cluster. The first one is the Euclidean 3D distance between the two points, the second one the deviation of their surface normal. After applying this region growing process, we obtain a set of clusters, which we subsequently feed to a plane-fitting algorithm. Once we have eventually found some clusters to be part of a plane, we can extract coplanar bases very easily, as any four points lying in the same plane are by definition coplanar. Following this procedure, we can omit the randomized base selection process of the original 4-PCS method. Obviously, it is still beneficial to pick wide bases (by selecting points that are located far from each other), as they are in general leading to more stable alignments. To this end, we prioritize points lying at the boundaries of the planar cluster to serve as a base. Of course, the base should still lie in the overlap region between the two point clouds in order not to miss the desired solution. As we are considering point clouds that are captured at more or less the same position (but at different moments in time), we assume that the overlap will be quite large. Only in the case that a large object close to the scanner is causing a huge occlusion in one of the point clouds, this assumption might be violated. Therefore, we propose computing for each planar region its convex hull and selecting a coplanar base by picking four points that are close to this convex hull. Figure 4 depicts two point clouds from sequence “05” of the KITTI benchmark together with the convex hulls of the estimated planar regions.

#### 4.2.2. Finding Congruent Sets

Once a coplanar base is selected, the next step consists of finding four-point sets in the other point cloud that are congruent to this base. This matching step is based on a specific property of affine invariants of four-point congruent sets. In a nutshell, given four points, we can compute two independent ratios between the line segments they are defining that are preserved under affine transformations. Given a set of coplanar points B={a,b,c,d} from point cloud P that are not all collinear. Let ab and cd be the two lines that intersect at an *intermediate* point *e*. The two ratios

(1) $$r_{1}=\frac{\left||a-e|\right|}{\left||a-b|\right|}\;r_{2}=\frac{\left||c-e|\right|}{\left||c-d|\right|}$$

are invariant under affine transformation and uniquely define four points up to affine transformations. Now, for each point $q_{1},q_{2}\in Q$, we can compute two *intermediate* points:

(2) $$e_{1}=p_{1}+r_{1}\left(p_{2}-p_{1}\right)\;e_{2}=p_{1}+r_{2}\left(p_{2}-p_{1}\right).$$

Any two pairs whose intermediate points $e_{1}$ and $e_{2}$ are coincident potentially correspond to a four-point set that is an affine transformed copy of B. Of course, as these four-point sets are the affine invariants of the base B, it is a superset of the four-point set that are a rigid transformation of the base. For that reason, we also check the angle between the two line segments to determine if the four-point set is a rigid transformation of the base B. Naturally, the intermediate points $e_{1}$ and $e_{2}$ will never exactly be coincident due to noise and other inaccuracies. Instead, they will end up on being *nearby* points. For that reason, we set up a k-d tree search data structure that allows for fast spatial queries. We then accept the set as being congruent to the base B in case the distance of the two intermediate points $e_{1}$ and $e_{2}$ are within δ-distance from each other. Another limitation of the original method of [26] is that it computes and tests for all possible combinations of points their intermediate points $e_{1}$ and $e_{2}$. This is leading to a tremendous amount of unnecessary computations. Instead, we propose only processing the points of clusters that are lying in a physical plane, i.e., a plane that is present in the scene. Only these points qualify as matchable with a given coplanar base, as the bases themselves were picked on the detected planar regions.

#### 4.2.3. Test Rigid Transformation

The final step in the 4-PCS method is to test the rigid transformation computed using the base and its congruent sets. One way of verifying the transformation is by using the *largest common point set* (LCP) criterion. This criterion states that one should count the number of points from the *source* point cloud that are within a δ-distance from any point from the *target* point cloud after alignment. The transformation that yields the largest LCP is considered the true transformation. We emphasize that we only compute the transformation and LCP criterion for the congruent sets that we have selected in the previous step. This group of congruent sets is thus a lot smaller than the original method proposed by [26] et al. Our algorithm can thus be summarized as follows. First, select a few strong coplanar bases from P based on the estimated planes in the scene. Second, given a selected coplanar base from P, determine only a few strong four-point sets in Q that are approximately congruent. For all these selected four-point sets, compute the transformation that aligns the two point clouds and eventually pick the transformation with the largest common point set. In other words, pick the transformation for which the most points of P are within δ-distance from a point in Q.

### 4.3. Verification through ICP and Geometrical Consistency

The 4-PCS method yields a rough transformation from source to target point cloud, but it will not perfectly align them. We therefore refine the transformation by using an ICP-based algorithm that was described in [24]. This ICP algorithm offers additional verification to conclude that the two point clouds yield a true loop. If the residual of ICP—defined as the average distance between all corresponding points—is too large, we still discard the loop candidate. Finally, the object clustering mentioned in the previous section also provides a means of verification, as we can check if all the object clusters are relatively still at the same position. To that end, we compute for each cluster its centroid and compute a bipartite matching using the Hungarian algorithm. If these two verification steps are positive, we can eventually proceed to the actual loop correction.

### 4.4. Loop Correction

As stated in the introduction, our goal is to implement loop closure as an online process. Therefore, we adopted a heuristic approach that is extremely fast, though leading to a sub-optimal solution. The idea is to avoid the iteration between the SLAM front- and back-end. The front-end refers to the scan matching process whereas the back-end deals with the global consistency of the 3D map and hence the correction of the loops. In that iteration, the outcome of the SLAM back-end, being the pose error, is given to the front-end to re-investigate its outcome, thereby taking into account all the known relations between neighboring poses and matched correspondences. After this, the outcome is fed back to the back-end to check if this re-investigation has led to a better result. This process is repeated for all poses and all correspondences, up until the optimal solution is found. It goes without saying that all this is inherently time-consuming, so we propose bypassing this iterative behavior. Instead, we pass the *local* information from the scan matching just once to the back-end, but not the other way round. In a nutshell, we investigate how much each pose is contributing to the final accumulated error, thereby considering the residual score of the scan matching process. The actual correction is then performed as follows. Consider the mapping platform travelling along the trajectory $V_{0},\ldots ,V_{i},\ldots V_{n}$, where at each pose $V_{i}$ the lidar scanner is capturing a point cloud $P_{i}$ during a full rotation of its head, also referred to as a sweep. We assume that the loop detection method provides us with two point clouds, i.e., the *start* and *end* of the loop. Let us denote their poses as, respectively, $V_{s}$ and $V_{e}$. Next, the difference in pose, i.e., the loop transform, is given by $\Delta ={\left(R_{s,e}V_{e}\right)}^{-1}V_{s}$. Note that the rotation $R_{s,e}$ denotes the one we have computed using our registration algorithm. It should be discarded from the loop correction process as it does not yield an error. This loop transform Δ is considered as an *error* as both poses $R_{s,e}V_{e}$ and $V_{s}$ should be equal. It should thus be projected back in the pose graph. As mentioned before, we use the residual of the scan matching process, i.e., final cost after transformation, to assign a weight $c_{i,j}$ to each edge in the pose graph. We assign a higher weight for those transformations that yield a high residual in the scan matching step. The idea is that a high residual indicates that two consecutive point clouds were potentially inaccurately aligned. In addition, we assume that the scan matching process will have already been converging in the right direction. Next, we define the *distance* between two poses $V_{k}$ and $V_{l}$ as $d\left(V_{k},V_{l}\right)=\sum_{i,j}c_{i,j}$. Herein, {i,j} denotes the set of all edges in the path from $V_{k}$ to $V_{l}$. Finally, we define a weight

(3) $$w_{i}=\frac{d(V_{s},V_{i})}{d(V_{s},V_{e})}$$

for each pose in the graph that specifies the fraction of the matrix Δ by which the pose has to be transformed. The poses $V_{k}$ are then updated replacing $t_{k}$ by $t_{k}w_{k}\Delta$ and $R_{k}$ by $slerp(R_{k},w_{k}\Delta )$, *slerp* denoting the spherical linear interpolation function as described in [29].

## 5. Evaluation

The evaluation covers three main parts. First, we conduct an analysis to qualify the speed-up of our GPU-accelerated descriptor computation. Second, we compare our loop detection accuracy to other state-of-the-art methods. Finally, the quality and the speed of the loop closure module are analyzed.

### 5.1. Speed Analysis Global Descriptor Computation

In order to compare our GPU implementation of the M2DP descriptor against the CPU version implemented in Matlab by [10], we ran several experiments using data from several Velodyne scanners. To indicate the performance on point clouds of different sizes, we used data from both the VLP-16, HDL-32E, and HDL-64E containing, respectively, 16, 32, and 64 lasers. More specifically, we used several sequences from the KITTI benchmark [30], which are all captured by an HDL-64E in urban environments. We extended this dataset with own recorded sequences in both indoor and outdoor scenes using the HDL-32E and VLP-16. The experiments were conducted on a computer with an Intel Core i7-7820X @ 3.60Ghz, 128GB RAM, and an nVidia GeForce GTX 1080ti inside. The results are summarized in Table 1. As can be seen, our GPU implementation scales well for larger point clouds. The overhead of copying data to the GPU memory is relatively low for larger point clouds, hence yielding a larger speed-up. For point clouds originating from the HDL-64E scanner, our implementation only takes 127 ms on average compared to 476 ms for the Matlab implementation of [10], which is a speed-up factor of nearly 4. For smaller point clouds—captured with the VLP-16 or HDL-32E—we notice a speed-up factor of almost 2.5.

### 5.2. Speed Analysis Global Registration

Regarding the speed of the global registration, we computed the alignment for all the *loop candidates* determined by the global descriptor for the KITTI sequences as well as for our own recordings in Ghent. We did this experiment for our own registration algorithm and for the original method of [26]. For all the experiments the value for δ was set to 0.5 m. This value is rather high, but due to the sparse point density, it turns out that this value is leading to the best results. The implementation of the algorithm of [26] also requires a parameter representing the number of “samples” taken per point cloud. This sub-sampling is necessary as the execution of the method is too slow when all points are used to select and match several bases. We have set the value to 2000 samples, as this turned out to lead to similar results compared to our implementation. The results are summarized in Table 2. It is important to note that our method and the one of [26] do not produce the exact same result, so the comparison of the computation time is purely indicative. As can be seen, the average processing time per alignment for our method is 184 and 159 ms for the KITTI and Ghent dataset, respectively. The KITTI dataset has been acquired with the Velodyne HDL-64E, hence consisting of four times as many lasers compared to the VLP-16E, but the computation time of our method is only 20% higher. The original method of [26] needs respectively 875 and 415 ms for the same datasets. Hence, for HDL-64E data, the original method needs approximately twice as much time to compute the alignment.

### 5.3. Loop Detection Accuracy

**Figure 5:**
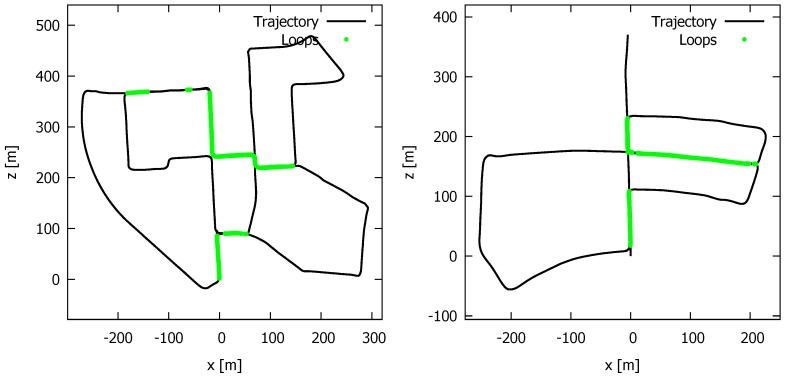
The ground truth loops in the “00” (**left**) and “05” (**right**) sequences of the Kitti benchmark [30]. We consider a loop to be present when the distance between two poses is less than 1 m.

In order to evaluate the accuracy of our *final* loop detector against the state of the art, we conducted several experiments on the Kitti benchmark [30]. More specifically, we used the sequences “00” and“05”, as these contain the most “revisited” locations. To generate ground truth, we used the known trajectories and considered a loop to be present when the distance between two poses was less than 1 m. Thereby, we used a threshold of 100 poses to prevent two subsequent poses from being wrongly classified as a loop. In Figure 5, the “ground truth” revisited locations in the two trajectories are depicted as green dots.

For some sequences, the same road was taken multiple times, so the whole part of the road was considered as a loop. However, as can be seen, sometimes there are green dots missing along a road that has been taken multiple times, meaning that the difference in pose is larger than 1 m. This could be due to the fact that some roads consist of multiple lanes and that a different lane was taken.

We used four different descriptors with which to compare our results. The first one was the original M2DP method described in [10]. The other descriptors were the *ensemble of shape functions* (ESFs) [31], spin images [6], and SHOT [7]. The latter two are local descriptors, so in order to use them as global descriptors we computed the centroid of the point cloud and estimated the spin image and SHOT descriptor related to this centroid. Thereby we used the maximum distance from the centroid to any other point in the cloud as the radius to compute the descriptor. Furthermore, both the spin images and the SHOT descriptors were based on normal vectors. To compute these, we used a radius that was five times the average distance of a point to its closest neighbor. Regarding spin images, the other parameters that needed to be set were (1) the number of bins along one dimension, (2) the minimal allowed cosine of the angle between the normals of the input cloud and search surface (for the point to be retained in the support), and (3) the minimal number of points in the support to correctly estimate the spin image. We set these parameters to, respectively, 8, 0.5, and 16. The SHOT descriptor did not have any other parameters to be set, and for the ESF we again used the maximum distance of the centroid to any other point in the cloud. The M2DP method needs the number of bins for each plane (expressed as the number of circles *l* and the number of bins in one ring *t*) and the number of planes to use (expressed as the azimuth *p* and elevation *q*). For our experiments, we set these values to l=8, t=16, p=4, and q=16 for all tests.

**Figure 6:**
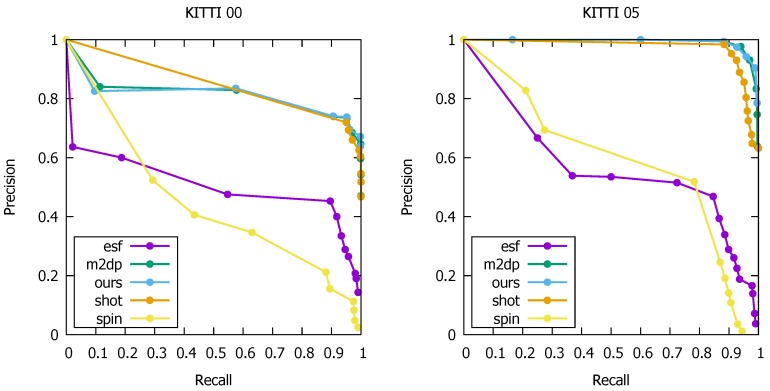
ROC curves for the several loop detection methods on the KITTI sequences “00” (**left**) and “05” (**right**). As can be seen, our method performs better than the original M2DP method, the second-best performer in our experiments. The SHOT detector also produces acceptable results, whereas the spin and ESF detectors are too unreliable to be used in practice.

The ROC curves for the different loop detectors are depicted in Figure 6.

We clearly see that our method along with the methods M2DP and SHOT lead to the best detections. The performance of the ESF descriptor and spin images turned out to be insufficient to reliably recognize revisited areas. To obtain a recall of at least 90%, the precision dropped to, respectively, 45% and 20% for the KITTI sequence “00”, which is unacceptable for an operational system. For the KITTI sequence “05”, the precisions corresponding to a recall of 90% were even worse, respectively, 30% and 15%. On the contrary, the M2DP and SHOT descriptors led to a precision of 70% on the “00”-sequence and were higher than 95% on the “05”-sequence for a 90% recall. In Table 3, the exact precision is listed that corresponds to a recall of about 99.9%. We observed that our combined method further improved the performance of the M2DP detector. For the KITTI sequence “05”, to obtain 99.9% recall, we reached a precision of 90.4%, an improvement of more than 7.1%. For the KITTI sequence “00”, we obtained a smaller improvement of 0.8%, reaching a precision of 60.5% compared to 59.7 for the M2DP detector. The lower performance on the KITTI “00” dataset was probably due to inaccuracies in the ground truth. A higher threshold on the distance for a pose to be considered as a ground truth loop affects the results tremendously. Thus, the value of this experiment is in the comparison between the different detectors rather than in the absolute numbers on the actual accuracy.

**Figure 7:**
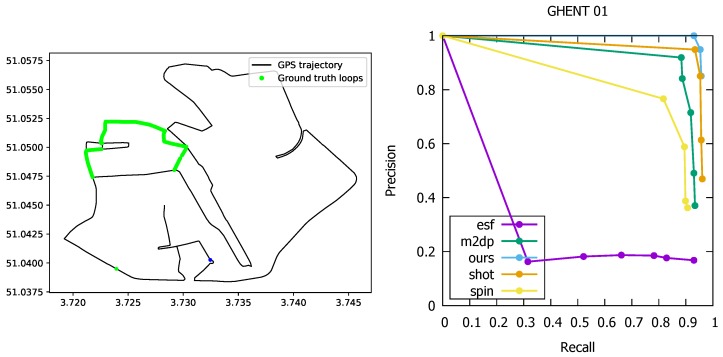
**Left:** The ground truth and estimated loops of the sequences recorded in the city center of Ghent, Belgium. We consider a loop to be present when the distance between two poses is less than 3 m. **Right:** The ROC curve for the sequence recorded in the city center of Ghent, Belgium. Our loop detector clearly outperformed the other methods. For a recall of 93%, we still obtained a precision of 100%, which is a 5.2% gain compared to the SHOT descriptor. The latter along with the M2DP descriptor were the only two other point cloud matchers that produced acceptable results. The loop detection quality of the spin images descriptor and ESF are too limited.

Besides experiments on the Kitti benchmark, we acquired a lidar sequence ourselves in the city of Ghent (Belgium), and mounted the Velodyne VLP-16 lidar scanner on top of a car. While acquiring the lidar data, we used a Garmin GPS to generate ground truth. The trajectory is shown in Figure 7. As can be seen, a part of the trajectory was taken twice, making all the poses along this part act as loops.

This time we used 3 m as a threshold for two poses to be considered as a loop, as this threshold led to more coherent loops along roads that were taken twice. As the sequence was recorded in the historical city center of Ghent, the GPS signal was often times inaccurate leading to a noisy trajectory. To deal with these anomalies, we used the Google API to *clean up* the trajectory by computing the most likely roads that were taken. This eventually led to 843 loops on a trajectory of approx. 15.7 km travelled in 47 min. The number of lidar point clouds was 31,745. For this experiment, we used the exact same parameters as those of the KITTI sequences. The ROC curve is depicted in Figure 7. In this experiment, our loop detector clearly outperformed the other point cloud matchers. For a recall of 93%, we still obtained a precision of 100%, which is an improvement of 5.2% compared to the SHOT descriptor. Only the latter along with the M2DP descriptor generated acceptable results. The spin images performed better than those for the KITTI sequences, but the overall accuracy is still too low to be used in an operational system. Finally, for the ESF descriptor, it was very difficult to achieve any acceptable precision, even for recall values. Due to inaccuracies in the GPS data, and hence the ground truth, it was not possible to reach 100% recall for any of the methods, as was the case for the KITTI.

### 5.4. Quality and Speed of the Loop Correction

**Figure 8:**
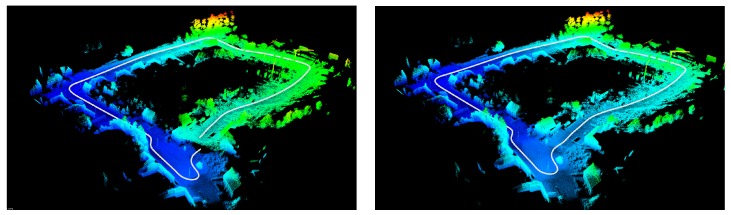
The two resulting reconstructions for the “Hasselt” dataset, before loop closure (**left**) and after loop closure (**right**). After loop correction, the two ends of the loops were attached, and the error was propagated back in the pose graph.

To assess the quality of our loop correction algorithm, we conducted an experiment using data that we captured in the Belgian city of Hasselt. We mounted a Velodyne HDL-32E on a mobile mapping van together with a high-precision POS-LV inertial positioning system to acquire accurate ground truth. In order to obtain the initial trajectory, we used our 3D reconstruction system described in [24]. In Figure 8, two images are depicted showing the result of this reconstruction process.

**Figure 9:**
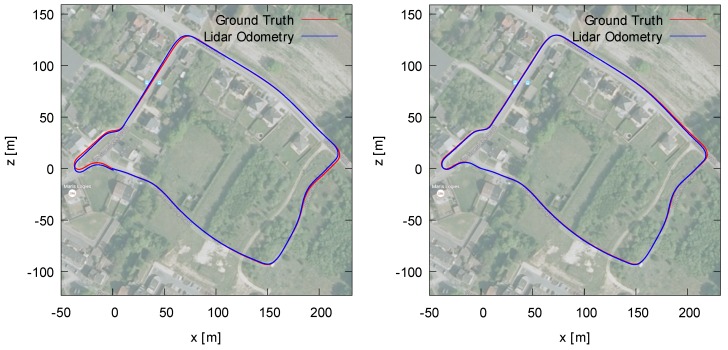
The two resulting trajectories for the “Hasselt” dataset corresponding to the reconstructions in Figure 8, before loop closure (**left**) and after loop closure (**right**). The estimated trajectory and the ground truth trajectory are almost entirely overlapping after loop closure. The pose error before and after loop correction is, respectively, 5.56 and 3.13 m, hence proving the effectiveness of the loop correction.

In the left image, the resulting point cloud is shown before loop closure, whereas the right picture depicts the point cloud after loop closure. As can be seen in the left point cloud, the end of the loop was not connected with the start of the loop. After correction, the two ends were connected, and the accumulated error was propagated back in the pose graph. In order to evaluate this quantitatively, we used the POS-LV positioning system as ground truth. Figure 9 depicts both the ground truth and the final trajectory after performing loop closure.

**Figure 10:**
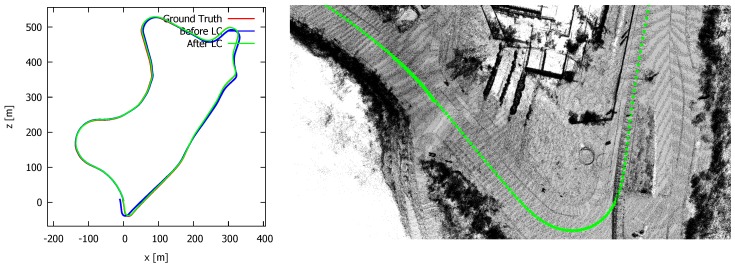
The resulting trajectory (**left**) of sequence “09” of the Kitti benchmark, before loop closure (blue) and after loop closure (green). The estimated and the ground truth trajectory are almost entirely overlapping after loop correction. (**right**) the corresponding reconstruction at the start- and end-point of the loop after correction. Little to no artefacts can be seen in the reconstruction. The pose error before and after loop correction is, respectively, 9.89 and 4.80 m, hence proving its effectiveness.

As we do not have a complete ground truth 3D model, we measured the quality by means of comparing all poses in the pose graph. To this end we computed the average distance from all poses in the estimated trajectory with its *closest* pose in the ground truth. The average distance between all poses before and after loop closure turned out to be, respectively, 5.65 and 3.13 m. Furthermore, the median pose error before and after loop closure were, respectively, 3.98 and 2.13 m. Hence, the loop closure process reduced the total error almost by a factor of 2. The total time to correct the loop on an Intel Core i7-4712HQ CPU @ 2.30 GHz was 11 ms. This is extremely fast thanks to the omission of the iteration between the SLAM front- and back-end. During the SLAM front-end, a residual score was computed that is later on used in the loop correction phase. The loop correction itself only involves one single manipulation of the poses. By means of a second example, we also closed the loop of sequence “09” of the Kitti benchmark. The result is shown in Figure 10. The trajectory after loop closure (in green) and the “ground truth” trajectory (in red) are almost entirely overlapping. In the right image, the point cloud at the start and end-point of the loop are shown. The poses are overlapping, and little to no artefacts can be seen in the point cloud. For this dataset, we also computed the average pose error before and after loop correction, which resulted in values of, respectively, 9.89 and 4.80 m. The median value before and after loop correction was 7.85 and 3.53 m, respectively. In summary, we can conclude that we reduce the error with a factor 2 after loop closure.

The results of both experiments are summarized in Table 4.

### 5.5. Speed Analysis Entire Loop Closure Process

In this subsection, we summarize the processing time for the entire loop closing process. It is important to note that, for most use cases, there will only be a few loops present in a sequence. Even in the case that an entire road is taken multiple times, it often suffices to close the loop only once or twice along that trajectory. Furthermore, the preliminary check for the presence of a loop is an extremely efficient operation: it just compares the current position with the previous ones, which only takes up to a couple of milliseconds. Only when the distance is smaller than 1% of the total distance travelled since the last loop closure, we consider it as a loop candidate and only in that case we compute the signatures of both point clouds. Based on the difference in signature, we decide whether or not we compute the alignment between them. This decision is dependent on a threshold which also affects the precision, recall, and computation time of the system. It should thus be properly fine-tuned. In the previous sections, we showed that the execution time for the alignment process is approximately 150–200 ms. Together with the 100–130 ms for the global descriptor computation, we can conclude that the entire loop detection process leads to a maximum overhead of about 250–350 ms per pose. In most cases, where there is no loop present, the real overhead is, however, just a couple of milliseconds to perform the preliminary check. Finally, when a loop needs to be closed, this causes an additional overhead of approximately 10–20 ms.

## 6. Conclusions

In this paper, a full loop detection and correction method is presented. The main contributions of this work are threefold. First, we accelerated the loop detection process by developing a GPU-accelerated version of a global feature descriptor for point clouds. Second, we present a novel registration technique to align two point clouds with a large deviation in orientation, and this method outperforms existing techniques regarding robustness, accuracy, and speed. We explain how the method can be used to align two point clouds that serve as the two ends of a loop in lidar data. Furthermore, we show the effectiveness of incorporating this registration technique in the verification process of loop candidates. Experiments demonstrated that we can gain up to a 7% precision for the same recall value of 99.9%. Experiments also showed that our method works for data acquired with different Velodyne scanners, including the one containing 64, 32, or 16 lasers. In addition, we show that our global feature descriptor is a factor of 2–4 times faster than the original version, depending on the number of points in the point cloud. Finally, we present a novel loop correction heuristic that reduces the average and mean pose error—defined as the distance of a pose with its closest neighbor in the ground truth trajectory—by a factor of 2.

## Figures and Tables

**Table 1:** Computation times of our GPU implementation of the M2DP descriptor compared to the CPU version implemented in Matlab by [10]. The speed-up factor for data originating from the HDL-64E is almost ×4, whereas for the VLP-16 and HDL-32E the speed-up factor is ×2.5.

Sensor	avg. |P|	Matlab ([10]) in ms	GPU (ours) in ms
VLP-16	25,446	250	**110**
HDL-32E	51,687	273	**121**
HDL-64E	62,594	476	**127**

**Table 2:** Processing times for our registration algorithm and the one of [26] on the KITTI dataset as well as on our own dataset recorded in Ghent.

Dataset	Sensor	δ	Time (ms) Ours	Time (ms) [26]	Num. Samples in [26]
Kitti	Velodyne HDL-64E	0.5	184	875	2000
Ghent	Velodyne VLP-16	0.5	159	415	2000

**Table 3:** Precision at 99.9% recall of the different loop detection methods for the KITTI dataset. Clearly, only the detectors SHOT and M2DP produce “acceptable” results. Our combined algorithm improves the original M2DP method by 0.8% and 7% for KITTI sequences “00” and “05”, leading to a precision of, respectively, 60.5% and 90.4%.

Sequence	Spin Image	SHOT	ESF	M2DP	Ours
KITTI00	0.025	0.575	0.143	0.597	**0.605**
KITTI05	<0.01	0.632	0.037	0.833	**0.904**

**Table 4:** The average and median pose error before and after loop closure for the Hasselt and Kitti “09” sequence. Our correction algorithm reduces the error by a factor of approximately × 2 after closing the loop.

Sequence	Total Length (m)	Average Error (m)			Median Error (m)		
		before LC	after LC	gain	before LC	after LC	gain
HASSELT	715	5.65	3.13	**×1.81**	3.98	2.13	**×1.87**
KITTI09	1705	9.89	4.80	**×2.06**	7.85	3.53	**×2.22**
